# Organizational Readiness Tools for Global Health Intervention: A Review

**DOI:** 10.3389/fpubh.2018.00056

**Published:** 2018-03-02

**Authors:** James W. Dearing

**Affiliations:** ^1^Michigan State University, East Lansing, MI, United States

**Keywords:** organizational readiness tools, global public health, organizational capacity, organizational motivation, implementation science, scale up

## Abstract

The ability of non-governmental organizations, government agencies, and corporations to deliver and support the availability and use of interventions for improved global public health depends on their readiness to do so. Yet readiness has proven to be a rather fluid concept in global public health, perhaps due to its multidimensional nature and because scholars and practitioners have applied the concept at different levels such as the individual, organization, and community. This review concerns 30 publically available tools created for the purpose of organizational readiness assessment in order to carry out global public health objectives. Results suggest that these tools assess organizational capacity in the absence of measuring organizational motivation, thus overlooking a key aspect of organizational readiness. Moreover, the tools reviewed are mostly untested by their developers to establish whether the tools do, in fact, measure capacity. These results suggest opportunities for implementation science researchers.

Despite a common emphasis on the development of effective global health interventions, the greatest contemporary challenges in improving the health of populations rest with the delivery and utilization of interventions (1). Delivery relies on many human factors—such as communication, coordination, training, leadership and management, logistics, transportation, storage, and community outreach and behavioral campaigns—that function both independently of and interdependently with technical systems. Delivery is necessarily reliant on *systems*, comprised of different types of organizations, their histories, and current ways of working together. Strengthening these systems and the organizations that comprise them represents a global health priority (2). In some topical areas such as maternal, newborn, and child health, the availability of effective and simple interventions has shifted the challenge of achieving impact at scale from the development of new interventions to the delivery and uptake of these evidence-based interventions (3, 4).

Delivery is achieved through systems which commonly function as partnerships between governments (e.g., ministries of health), the non-profit sector, and private industry. Especially when pursued at large scale, delivery demands a degree of readiness to implement interventions, which reflects both organizational abilities and a desire to affect change (5).

In this article, I review the state of applied tools for assessing organizational readiness for global health intervention, and suggest how they might be improved through research and evaluation.

## Readiness Means Motivation Combined with Capacity

The term *readiness* has often meant a psychological state (if measured at organizational and community levels, this represents a shared “state”) of commitment to a particular course of action (6). For example, individual readiness can refer to a person’s resolve to stop smoking, organizational readiness can be a shared belief by hospital staff that hospital acquired infections are unacceptably frequent, and community readiness may be represented by the degree to which community leaders are supportive of an effort to share patient health record data across competing health clinics. For global health work in less-developed countries, assessing the degree of *motivation* of organizations that are candidates to deliver or implement interventions such as bed nets to prevent malaria, biomedical interventions such as pre-exposure prophylaxis for HIV prevention, or inexpensive and clean burning cook stoves is important since many NGOs, aid organizations, and government agencies work on multiple challenges at once and sometimes relegate some interventions to a low priority. So success requires the presence of an important attitudinal component; organizations need to be appropriately motivated or willing for the organization to engage in a particular intervention (7–9).

In addition to motivated organizations, successful global health intervention in less-developed countries requires those organizations to have the skills, training, and resources to do a good job. *Capacity* is the ability to carry out stated objectives (10). The concept includes both the ability to produce an output, such as a functional community health outreach worker program, and the effectiveness of those outputs to produce desired health outcomes. Outcomes represent performance: How well do an organization’s activities induce the desired effect on outcomes such as individual behaviors and community or population health?

The concept of capacity has proven rather fluid in global health, perhaps due to its multidimensional nature and because scholars and practitioners have applied the concept at different levels; for example, *capacity* has been used to describe individual, team, organizational, and community abilities. At any level of analysis, capacity is also subject to exogenous inputs such as policy decisions and funding availability (11). As a result, the extent to which organizational capacity and outputs are responsible for observed outcomes is often difficult to accurately determine. This is one reason why investments in organizational capacity building (or when raised a level, system strengthening) can be controversial (12).

So organizational readiness assessment can be performed to (1) learn about the degree of motivation within a candidate organization for delivering and implementing a global health intervention, (2) assess the particular abilities within organizations, (3) help improve one or more organizational capacities, or (4) empower organizations to bring more value to their clients. Each objective can be useful. For example, a funder may want to compare which of a set of non-governmental organizations is best suited to deliver mosquito nets, conduct radio campaigns about them, and train community outreach workers in their correct use, with no intent to affect organizational capacities. Or an organizational leader may want to understand her organization’s capacities in order to set improvement or budgetary priorities. Or a policy maker may realize that a grassroots community-based organization has yet to fully develop its technical skills, but has strong and authentic access into those communities; thus, assessment can be used to help organizations from marginalized populations to better understand health risks and deliberate over alternative solutions, as well as help those organizations to work effectively with and for the community stakeholders they represent (13).

This review presents and analyzes readiness assessment tools that purportedly measure organizational readiness. We began with a systematic search of published and unpublished (gray) literature for tools (including decision aids and instruments such as questionnaires) that were designed to provide information about the capacity and motivation of organizations involved in global health.

## Inclusion Criteria and Methods

For this review, publically available resources must have had:

- Addressed *organizational* capacity and/or motivation (tools that assessed general organizational assessment or performance were not included)
- Been relevant to the objectives of global health interventions (resources primarily assessing or addressing system- or individual-level factors were not included)
- Been created for organizations working in *global health or international development in low-resource countries*
- Contained a *tool, instrument*, or *decision aid* that:
  ⚬ Facilitates *decision-making*
  ⚬ Provides recommended *measures* or operationalized frameworks/questions to assess capacity and/or motivation
  ⚬ Enables *quantitative or qualitative* measurement of factors

We defined *factor*s as attributes thought to contribute to organizational motivation and capacity to perform a service or function. A *tool, instrument*, or *decision-aid* was defined as a published collection of measures or factors meant to assist individuals in assessing the capacity or motivation of an organization.

Resources were identified using a systematic search of computerized databases (OVID, Web of Science, Academic Search Premier, CSA Sociological Abstracts), in-depth web-based searches, and bibliographic snowballing and back referencing. These search strategies were supplemented with targeted searches of relevant organizations, including World Bank Institute (WBI), the United Nations Development Programme (UNDP), John Snow International (JSI), United States Agency for International Development (USAID), and the UK Department for International Development (DFID), among others.

Our search identified 141 potentially relevant tools. An initial review was conducted by at least two members of the research team to assess each tool against the inclusion criteria. This process was done by reviewing the web sites for each tool and then the instructions and items specific to each tool. Each tool was assessed against each inclusion criterion. Tools were independently reviewed by two trained coders and coded for a range of variables. Differences of opinion were resolved through discussion and, in instances of continued disagreement, by the project manager who had trained the coders.

## Results

Thirty tools met the inclusion criteria. These 30 tools are included in this analysis (see Table 1).

**Table 1:** Organization capacity assessment tools (*N* = 30).

Tool name	Developed by
Assessing Management Capacity Among Non-governmental Organizations	CARE International

Capacity Assessment Framework	United Nations Development Programme (UNDP)

Capacity Building in Training-Dimensions and Indicators	PRIME/INTRAH

Capacity Development Results Framework (CDRF)	World Bank Institute (WBI)

Discussion-Oriented Organizational Self-Assessment (DOSA)	USAID Center for Development Information and Evaluation

Dynamic Participatory Institutional Diagnosis (DPID)	Senegal PVO/NGO, New TransCentury Foundation, Yirawah International

How to Assess NGO Capacity?	Norwegian Missionary Council Office for Development Cooperation

Institutional Development Framework (IDF)	Management Systems International (MSI)

Institutional Self Reliance (ISR)	Research Triangle Institute for UNDP

Management and Organizational Stability Tool (MOST)	Management Sciences for Health

McKinsey Capacity Assessment Grid/Effective Capacity Assessment for Non-Profit Organizations	McKinsey, Venture Philanthropy Partners

NGO Capacity Analysis	International HIV/AIDS Alliance

NGO Sustainability Index	USAID Office of Democracy and Governance; USAID Bureau for Europe and Eurasia

Nonprofit Organizational Assessment Tool-Strategic Planning Assessment Tool	Andrew Lewis, University of Wisconsin Extension

Organizational Capacity Assessment (OCA) Tool	PACT

Organizational Capacity Assessment Tool	Marguerite Casey Foundation

Organizational Capacity Audit Tool	GeSCI (Global e-Schools and Communities Initiative)

Organizational Capacity Indicator (OCI)	Christian Reformed World Relief Committee (CRWRC)

Organizational Capacity Self-Assessment Tool	Academy of Educational Development (AED), Croatia’s Non-governmental Sector (CroNGO)

Participatory Organizational Evaluation Tool (POET)	PACT, United Nations Development Programme (UNDP)

Partner Assessment Form	The Partnering Initiative/International Business Leaders Forum (IBLF)

Partner Organizational Capacity Assessment: A Tool for Assessing and Building Capacity for Twinning Partnerships for High-Quality Response to HIV/AIDS	Twinning Center

Partnership Self-Assessment Tool	Center for the Advancement of Collaborative Strategies in Health

Rapid Organizational Assessment	Universalia

Simple Capacity Assessment Tool (SCAT)	Education Development Center, PACT

Tool to Assess Site Readiness for Initiating Antiretroviral Therapy (ART) or Capacities for Existing ART Sites	John Snow, Inc. (JSI)

Training and Technical Assistance Plan (TTAP)	Counterpart International

UNDP CAPBUILD User’s Guide	United Nations Development Programme (UNDP)

Unity Foundation Capacity Quotient: A Diagnostic Tool for Benchmarking Capacity	Unity Foundation

USAID/Madagascar Institutional Capacity Questionnaire	USAID

The 30 tools are of different types (decision trees, questionnaires, checklists, matrices, etc.) and formats (paper, mobile, web-based, etc.) as listed in Box 1. Tools had a mean number of 60 questions or items grouped into a mean number of 13 factors.

Box 1 **Types and formats of tools.**

**Table:** 

Tool types (types can be >1)	(*N*) Tools
Self-assessment	24
External-assessment	10
Questionnaire	15
Matrix/grid	6
Guideline	1
Decision tree	1
**Tool formats**	
Paper-based	26
Web-based	2
Electronic spreadsheets	2

All tools addressed capacity; none addressed motivation. Each tool was coded for all factors it addressed and/or measured. Our team inductively developed a composite matrix of the capacity factors represented in the tools. Coders had been trained to familiarize themselves with barriers to or facilitators for the scale up of global health interventions in low-income countries (14–18). We then conducted an iterative analysis to identify those domains and factors most commonly addressed in the 30 tools. We grouped the factors into five domains associated with organizational capacity: (1) External Environment; (2) Organizational Attributes; (3) Management and Governance Capacity; (4) Collaboration; and (5) Organizational Performance. Each domain contains between two and eight factors (see Table 2). Analysis was completed based on totals by domain and factor, and by tool.

**Table 2:** Categorization of tool domains and factors.

**Organizational attributes**
Financial resources
Human resources
Infrastructure
Internal communication, knowledge management, and organizational learning
Leadership
Mission and vision (mission, strategy, organizational fit)
**Management and governance**
Adaptive capacity
Administration and organizational structure
Financial resource management
Human resource management
Measurement and evaluation
Strategic management
**Collaboration**
External partnerships and communication
Stakeholder partnerships
**Organizational performance**
Delivery, procurement, and supply
Institutional sustainability
Outputs, service, and results
**External environment**
Political/legal environment (including advocacy)
Sociocultural and geographic environment

The tools reviewed here assessed capacities of organizations by allowing users to enter qualitative and/or quantitative data, derived from expert judgment, interviews with staff and stakeholders, document review, workshops, and observation. Fourteen tools allowed both qualitative and quantitative data, 11 allowed only quantitative input, generally in the form of ordinal scales, and 5 allowed only qualitative input. Eleven tools provided scores or ratings by capacity factor and/or a composite capacity score or rating. A few tools also provide graphic output to provide a visual comparison of reported organizational strengths and weaknesses. Of the 30 tools, half addressed at least 40% of the factors identified in our review.

Of the five domains, Organizational Attributes, Management and Governance, and Collaboration were most consistently represented in the tools; 67% of tools included at least one factor in the Organizational Attributes and/or the Management and Governance domain. Seventy-three percent of tools included a least one factor in the Collaboration domain.

The Organizational Attributes domain includes tangible resources belonging or accessible to an organization (e.g., human, financial, technical, infrastructure), as well as intangible resources, such as the organization’s goals, knowledge, work and funding history, and culture. Approximately two-thirds of tools addressed these factors. Of these, mission and vision, human resources, financial resources, and infrastructure were most commonly addressed. Communication, leadership, and organizational culture are also commonly measured.

The Management and Governance domain addresses those systems, structures, and processes needed to effectively manage an organization. Financial management factors were most commonly addressed; also commonly measured were strategic management and administration, organizational structure factors, human resource management and measurement and evaluation.

Collaborations include relationships and communication with external partners—including governmental agencies, potential partner organizations, and stakeholders. These two factors appeared concurrently in more than half (57%) of all tools reviewed.

The External Environment and Organizational Performance domains were less consistently represented. Within the External Environment domain, 60 and 27% of tools operationalized political–legal/economic and sociocultural/geographic factors, respectively; 23% of tools addressed both factors and 37% did not include either factor. Forty percent of tools did not include any factors within the Organizational Performance domain; only 3% of reviewed decision aids included all three factors within this domain. Box 2 lists those capacity factors that are most prevalent in the 30 tools.

Box 2 **Top 10 capacity factors.**

**Table:** 

Factor	% of tools that measure this factor (*N*)
Mission and vision	87% (26)
Financial management	83% (25)
Financial resources	80% (24)
Strategic management	80% (24)
Administration and organizational structure	80% (24)
Human resources	73% (22)
External partnership and communication	73% (22)
Stakeholder partnership	73% (22)
Human resource management	67% (20)
Infrastructure	63% (19)

## Discussion

Assessing the readiness of organizations for global health intervention purposes should, according to the literature, involve measurement of both the capacity and the motivation of those organizations to engage in initiatives. Yet this review found only capacity assessment instruments. Measurement of an organization’s motivation or willingness to prioritize and engage in a global health intervention should be included and made available in such tools either in combination with existing capacity assessment tools or as stand-alone instruments.

Organizational capacity assessment tools with relevance for global health intervention measure many of the same domains and factors. This similarity may reflect a tendency by tool developers to employ common frameworks or orientations about what constitutes organizational capacity for global health intervention. It is possible that tool developers used a common evidence base to derive the measures included in the tools. These observed similarities may highlight an emerging consensus and convergence in scope regarding those factors that predict organizational effectiveness in the delivery of global health interventions.

The tools included in this review are focused at the organizational unit of analysis. As more organizational alliances active in efforts to scale-up health impact emerge, the most relevant level of analysis for estimating likelihood of success will be at the inter-organizational system or partnership level, reflecting the necessity that entire supply chains of collaborating and contracted organizations are involved when interventions are to be delivered to millions of people across large geographic areas (19). At this juncture, we did not find any tools that conceptualized and operationalized capacity or motivation measures at the level of inter-organizational systems or partnerships. While it can be expected that, because systems and inter-organizational partnerships are comprised of organizational actors, existing measures of organization-level capacities should be relevant, it is also the case that systems and partnerships require greater attention to working with heterophilous others (e.g., ministries of health, private organizations, non-governmental organizations, community health outreach workers). This requires coordination and contracting, and working across organizational boundaries where there may not be systems in place to seamlessly support such large-scale initiatives. Capacity assessment tools do not currently reflect the special challenges of this aggregate level of agency. Yet they could by focusing each partner organization’s experiences with the other organizations, for example, and measures focused on identification of complementary skills and resources across organizations.

## Do Organizational Readiness Assessment Tools Work?

Given the current information available, we cannot conclude whether these assessment tools are effective. Many highly reputable organizations have sponsored the development of these tools; perhaps these tools have been broadly and enthusiastically applied in practice to good effect. Yet we were unable to find data for any of the tools regarding validity assessment or utilization evaluation. None specifically presented evidence supporting the inclusion or validity of specific measures. In general, it appears that developers have relied on expert opinion and structural measures—with an unproven relationship to outcomes—when developing indices and measures. We do not know if use of any of these tools is associated with improvement in organizational motivation or capacities, initiative performance, or efficient use of resources. Neither did we find information about how much any of these tools has been used.

The partial convergence of these tools on similar factors related to capacity suggests that these instruments tap into correct constructs. That is, the factors most commonly assessed are likely meaningfully related to organizational capacity. A number of the 21 factors in this review are well reflected in the literature about scaling up in low-income countries (17, 20). However, improvement and refinement of the composition of items contained in these tools is probably possible.

## Utilization Considerations

Best practices for instrument and heuristic development suggests that tools or decision aids should be actively tested, refined, and improved with plausible potential users during a pre-testing stage conducted prior to release of the tool. Among other things, stakeholder feedback to prototype tools can provide exceedingly valuable insights into format preferences, optimum length, question order effects, response variance, use of graphics, and which types of potential users are best suited to provide different types of information. Though this may have occurred during development of the reviewed tools, the information available to us rarely included detail about such user-facing formative evaluation. Furthermore, stakeholders can differ considerably in their preferences for applied tools. Yet none of the 30 tools reviewed here was available in more than one interface or format.

Some tools reviewed here were developed in the 1990s, prior to remarkable developments in web-based applications. Nevertheless, the paucity of interactive capacity assessment tools for global health stakeholders is striking. It is likely that many users of these tools would find value in an instrument that is able to provide computations, comparative assessments, confidence intervals, or qualitative but tailored feedback based on data entered by the user. Of course, the utility of interactive formats would be limited to users with access to computers or electronic devices with internet connections.

An important implication for utilization lies in the understanding that much of the value in tools such as these lies beyond the data provided by the user and/or as computational or informational output. The process of engaging in use can be quite valuable for purposes of critical reflection, discussion, the development of a shared understanding among stakeholders, and the stimulation of a collective organizational will to improve processes and outputs. This type of *enlightenment use* has been shown in some research to be of more consequence in terms of learning and organizational improvement than is the instrumental data (the “answers”) themselves (21).

## Next Steps in Tool Development

A previously published framework used to evaluate measurement systems in public health (22) employed four criteria to assess instruments:

- Clarity of measurement parameters and normative standards
- Balance between structural and process measures
- Evidence for effectiveness
- Specification of an accountable entity

These criteria, when applied globally to currently existing organizational capacity assessment tools, highlight challenges and opportunities for improvement. Many of the tools we reviewed employ clear standards; the best quantitative scales are tied to well-defined categories that reflect the continuum of organizational development. The newer and more robust tools strike a balance between structural and process measures, as well as specification of accountable entities.

This review suggests a need for robust, validated, and user-friendly tools to measure organizational capacity and, we suggest, organizational motivation; taken together, such tools can more fully represent the construct of organizational readiness for global health intervention. Identified strategies for instrument improvement include standardization, evaluation, validation, and application of an evidence base to inform tool development. This evidence base could, in part, be constructed using retrospective case studies of how global health intervention delivery fared to assess whether successes and failures were associated with certain factors. Information could also be gathered about the valence and weighting of those factors. An evidence base could then be applied prospectively, using predictive tests to determine whether tool use affects roll-out or scale-up of global health interventions, and how. These research validation steps, if applied in tandem with utilization study during formative development, could result in tools that not only work but also work well for users. As the evidence base supporting the identification of core domains, factors, and appropriate methodologies evolves, tools such as these will become more valid, reliable, and useful for the increasingly diverse range of stakeholders involved in global health interventions.

## Author Contributions

The author originated the project and drafted and rewrote the manuscript.

## Conflict of Interest Statement

The author has no commercial conflicts of interest regarding any of the assessment tools reviewed.
